# Association Between Inclusive Educational Environments and Food Allergy Comprehension Among Preschool Children: A Quasi-Experimental Digital Intervention Study

**DOI:** 10.3390/nu18182972

**Published:** 2026-09-11

**Authors:** Tomoko Noma, Kyosuke Nowaki, Mei Nunoya, Haruna Koyama, Ayano Nishizoe

**Affiliations:** 1 Department of Health and Nutrition, Hijiyama University, 4-1-1 Ushitashin-machi, Higashi-ku, Hiroshima 732-8509, Japan; 2 Department of Nutrition, Koshien University, 10-1 Momijigaoka, Takarazuka-shi 665-0006, Japan; nowaki@koshien.ac.jp (K.N.); m-nunoya@koshien.ac.jp (M.N.); harugram1112@outlook.jp (H.K.); ayano14125@yahoo.co.jp (A.N.)

**Keywords:** ARCS model, digital nutrition education, food allergy, inclusive education, preschool children

## Abstract

**Background/Objectives:** This study examined whether sharing a class with a peer with physician-diagnosed food allergy (FA) was associated with post-intervention FA comprehension among preschool children. **Methods:** A four-month ARCS motivation model-based digital nutrition education program was delivered to 163 five-year-old preschoolers from nine public kindergartens. The program provided instruction on knowledge of balanced diets and FA. Preschoolers were classified as inclusive (at least one peer with physician-diagnosed FA in the same class; n = 75) or non-inclusive (n = 88). Dietary balance and FA comprehension were assessed using illustrated worksheets. Ordinal logistic generalized estimating equations (GEE) accounted for clustering within kindergartens. **Results:** The inclusive environment was associated with higher FA comprehension (Wald χ^2^(1) = 4.750, *p* = 0.029; OR = 4.219, 95% CI: 1.156–15.399), whereas no association was observed for dietary balance (Wald χ^2^(1) = 0.053, *p* = 0.818; OR = 0.912, 95% CI: 0.414–2.008). **Conclusions:** The presence of a peer with FA was associated with higher post-intervention FA comprehension.

## 1. Introduction

The increasing prevalence of food allergies (FA) is posing a significant public health issue [1,2]. In developed countries, approximately 10% of the population has some form of FA [2,3]. In Japan, a similar upward trend has been observed [4], with infants and preschoolers aged 0–6 years accounting for approximately 74% of all patients with FA [5]. Children with FA face not only physical health risks from accidental ingestion but also psychological burdens, such as a sense of stigma or the fear of being a “burden” to others [6,7,8,9,10,11,12]. Therefore, early childhood education that fosters a correct understanding of FA and empathy among peers is essential for creating a safe and inclusive environment.

Underlying this learning process, “observational learning” and “vicarious experience”—key concepts from Bandura’s social cognitive theory [13]—may play important roles. In other words, by observing the actions and experiences of others, children can acquire knowledge and social behaviors even without direct personal experience [14,15]. Related perspectives on situated and experiential learning likewise emphasize that knowledge can be shaped by participation in everyday contexts and by opportunities to connect new information with familiar experiences [16,17].

In a classroom that includes a child with FA, peers may encounter FA-related information through multiple pathways, such as everyday classroom routines, teacher guidance, or interactions with classmates. Such contextual exposure could potentially make later FA-related information more familiar or meaningful. However, the frequency and nature of these experiences, including observational learning itself, have not been well characterized in preschool settings [18,19,20].

In this study, we hypothesized that preschoolers in an inclusive educational setting would demonstrate higher comprehension of FA following the digital nutrition education intervention. To test this hypothesis, we first developed a digital nutrition education DVD based on the ARCS motivation model [21,22], proposed by Keller to enhance learning motivation. The ARCS model classifies learning motivation into four components—Attention, Relevance, Confidence, and Satisfaction—and has been successfully applied in early childhood learning and health education [22]. The use of digital educational materials that capture young children’s interest is gaining attention as an effective intervention method [23,24]. We then evaluated the comprehension level of dietary balance and FA following the DVD-based digital nutrition education intervention in both inclusive and non-inclusive educational settings.

## 2. Materials and Methods

### 2.1. Research Design and Feasibility Study

This study employed a non-randomized quasi-experimental design. Prior to the main study, we conducted a preliminary study in March 2021 at 10 public kindergartens in T City, Hyogo Prefecture, Japan, using an original nutrition education DVD. We assessed the feasibility of the intervention, including its practicality and acceptability in kindergarten settings, through observations of children’s engagement and teacher questionnaire responses (Table S1).

### 2.2. Participants and Implementation Procedures

The main intervention was conducted from November 2023 to February 2024 in nine public kindergartens in T City. The nine participating kindergartens had 188 enrolled five-year-old children. The digital nutrition education program consisted of four monthly sessions, each lasting approximately 25 min. In March 2024, 163 children completed the comprehension assessment and were included in the analysis. Individual attendance records for each of the four educational sessions were not kept. Based on information provided by kindergarten teachers, children were classified according to whether there was at least one classmate in the same class who had been diagnosed with FA by a physician. The inclusive group consisted of 75 children from five kindergartens, while the non-inclusive group consisted of 88 children from four kindergartens. In this municipality, FA requiring management in kindergarten is documented using medical records such as the “School Life Management Certificate” [25], and teachers use these records to identify children diagnosed with FA by a physician (Figure S1).

### 2.3. Digital Nutrition Education Program

The intervention utilized four original DVDs developed in accordance with the ARCS model. The educational content was designed to provide fundamental knowledge about FA and dietary balance. To teach balanced diets, we adapted the framework of the Japanese Food Spinning Top (Figure S2), a national nutrition education tool developed by the Ministry of Health, Labour, and Welfare and the Ministry of Agriculture, Forestry, and Fisheries [26]. The guideline uses a spinning top image and quantitative “Serving” units, which we simplified to match the developmental stage of five-year-old children. Instead of quantitative metrics, our program focused qualitatively on the importance of consuming items from all five core food categories: grain dishes, vegetable dishes, fish and meat dishes, milk and milk products, and fruits. To facilitate easy understanding and memorization without the spinning top visual, we introduced an original pedagogical approach in which the five fingers of a hand represented the five food categories. The educational effectiveness of this approach has already been reported [27].

To standardize implementation, the same set of DVD materials was provided to all nine kindergartens through the T City Board of Education and distributed to kindergarten principals during the same period. The DVDs were designed to be self-contained and to proceed without additional teacher instruction; for example, the original characters introduced and guided the educational content. Kindergarten principals reported completion of the scheduled sessions to the Board of Education. In addition, the researchers visited one participating kindergarten and directly observed a session to confirm that children were attending to the DVD program as intended. Formal fidelity monitoring using standardized checklists or direct observation was not conducted at all nine kindergartens.

**Figure 1:**
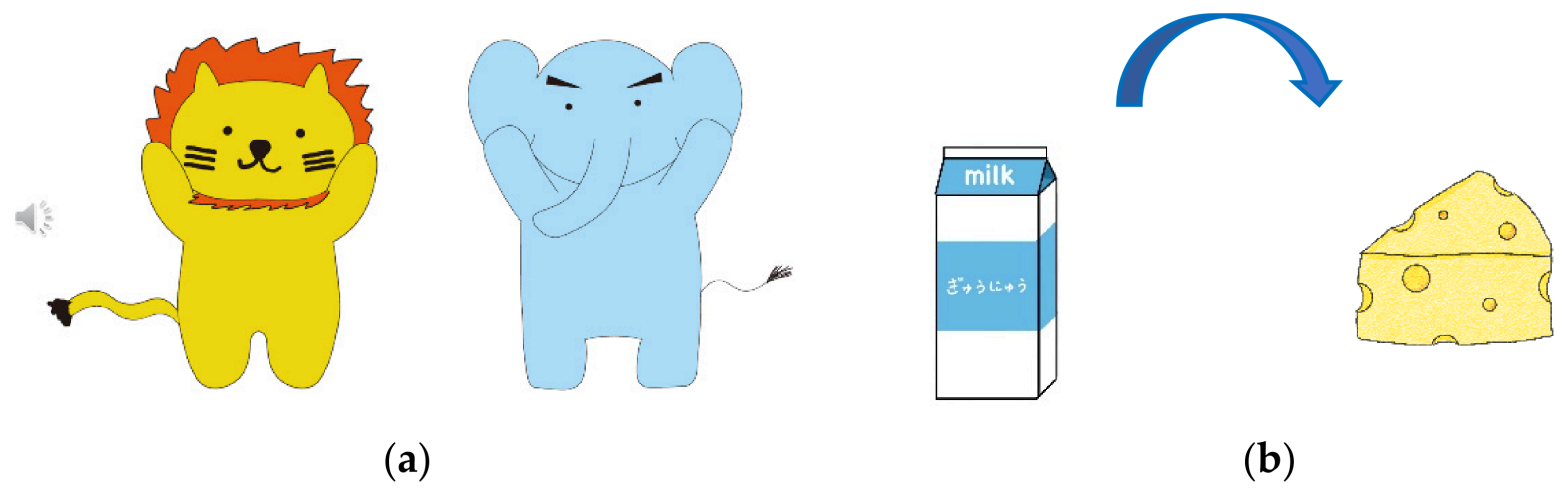
Contents of the Food Education DVD: (**a**) original characters used in the nutrition education DVDs; (**b**) an illustration explaining that cheese is made from milk.

**Figure 2:**
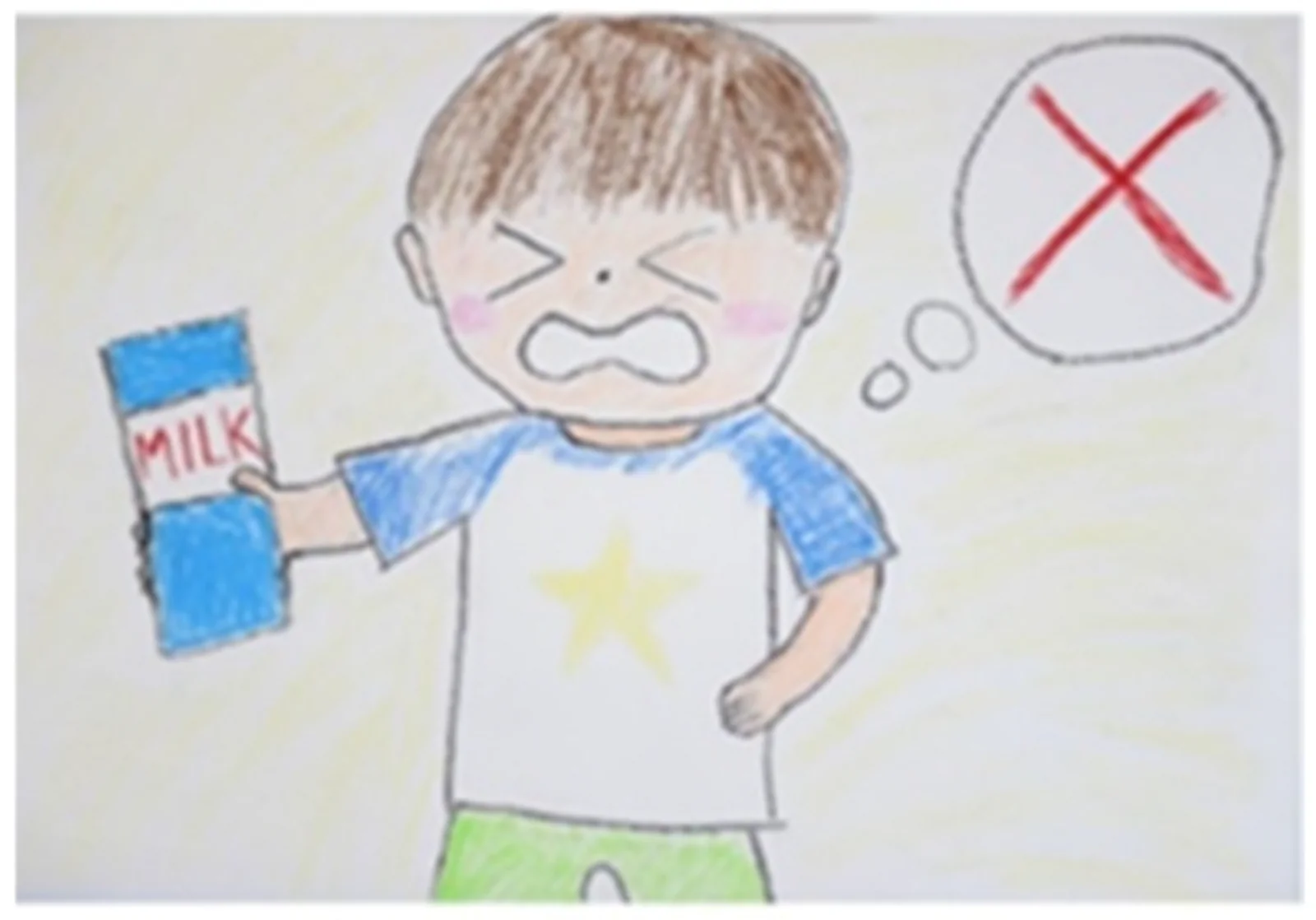
Contents of the Food Education DVD: a scene from a digital picture-story show explaining that children with FA may experience itching or stomachaches after drinking milk.

Based on findings from our preliminary feasibility study, we incorporated specific educational strategies in each ARCS component to optimize the learning process for five-year-old children:

- Attention: “A greeting from a character” was used to stimulate children’s situational interest and capture their initial attention at the beginning of each session (Figure 1).
- Relevance: “Quizzes” (Figure 1) and “digital picture-story presentations” (Figure 2) linked the educational contents (FA rules and the importance of eating a balanced diet) to the children’s daily lives, helping them perceive the topic as personally meaningful.
- Confidence: “Quizzes” and “dancing” were implemented to build self-efficacy. By answering Quiz Questions correctly and engaging in physical movement, children experienced small successes, thereby reinforcing their recognition and confidence in understanding FA and balanced meals.
- Satisfaction: “Quizzes” and “dancing” were also utilized to provide positive reinforcement. The program praised children’s correct understanding and participation, offering a sense of achievement and a natural reward for their learning efforts.

### 2.4. Assessment of Comprehension and Survey Methods

**Figure 3:**
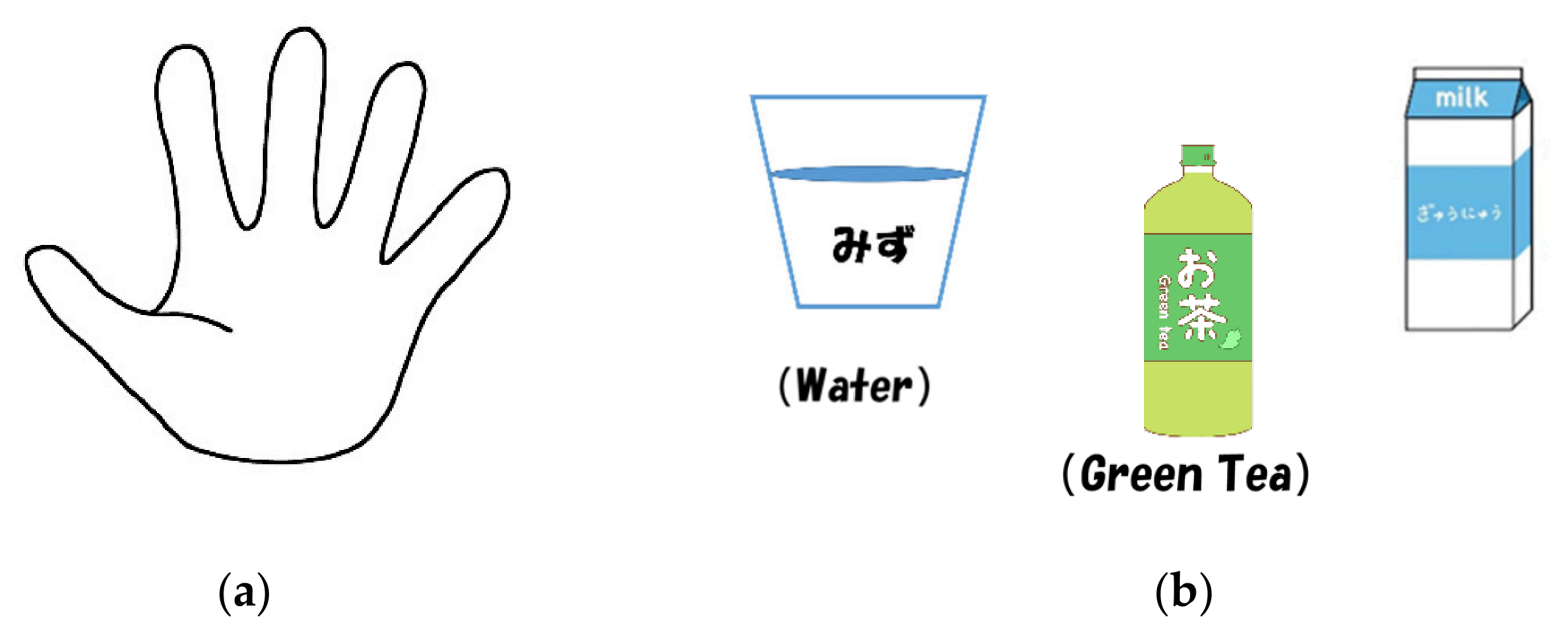
Assessment Worksheets: (**a**) A worksheet designed to assess understanding of a balanced diet. After conducting a nutrition education session on dietary balance, we showed the children various foods and asked them to use crayons to draw pictures on this worksheet indicating the finger to which each food corresponded. (**b**) A worksheet designed to assess understanding of FA. The children were asked to select foods related to allergies from the illustrations on the worksheet and circle them with crayons.

In March 2024, the researchers visited all nine kindergartens to conduct on-site assessments. The children’s level of understanding was assessed using illustrated worksheets (Figure 3) designed for children with limited reading skills. The assessments were conducted at each kindergarten and took approximately 15 to 20 min. We provided the oral instructions for completing the worksheets at all nine kindergartens and informed the children that they did not have to answer if they did not wish to participate. Regarding dietary balance, based on the “Dietary Balance Guide” that was introduced in the DVD, the children answered five questions by drawing pictures with crayons (1 point per question, total: 5 points). For FA, the children selected the correct answer from the illustrations on the worksheet and circled it with a crayon (1 point per question, total: 2 points). These two FA items were designed to concisely assess the children’s understanding of specific FA-related content presented in the program.

The worksheet questions were created by the researchers to correspond directly to the images and concepts presented in the teaching materials. The draft questions were submitted to the local board of education and kindergarten principals; feedback—with particular attention to appropriateness for the developmental stage of 5-year-olds, ease of understanding, and feasibility—was incorporated to create the final version. For the two FA comprehension items, the inter-item correlation was *r* = 0.473, and Cronbach’s alpha was 0.640. During the assessment, sufficient time was allowed for the children to answer the questions independently. The worksheets were completed anonymously and collected in a manner that prevented the identification of individual children.

The teacher questionnaire was administered anonymously and voluntarily to teachers involved in educational sessions for 5-year-olds at the nine participating kindergartens. Completed questionnaires were collected through the T City Board of Education. No information was collected to determine the total number of teachers eligible to participate in the questionnaire or number of respondents from each kindergarten. This questionnaire used a visual analog scale (VAS; 0–100 mm) to assess children’s participation and the teacher’s perceived effectiveness of the program (Table S2).

### 2.5. Statistical Analysis

Statistical analysis was performed using IBM SPSS Statistics version 31.0 (IBM Corp., Armonk, NY, USA), with a two-sided significance level set at *p* < 0.05. Since the participating children were nested within nine kindergartens, generalized estimating equations (GEE) were used as the primary analysis method to account for within-kindergarten correlations, with kindergarten ID specified as the clustering variable. The dietary balance score (0–5 points) and FA comprehension score (0–2 points) were treated as ordinal categorical outcomes. The non-inclusive group was designated as the reference category. Results were reported as Wald χ^2^ statistics, odds ratios (ORs), 95% confidence intervals (CIs), and *p*-values.

### 2.6. Ethical Considerations

This study was conducted with the approval of both the Koshien University Ethics Committee (Approval No.: R-4-2-2, Approval Date: 10 August 2023) and the T City Board of Education (Approval No. 950) with the cooperation of the participating kindergartens.

## 3. Results

### 3.1. Preliminary Survey (Feasibility Study)

The preliminary feasibility study indicated that the DVDs could be screened in the participating kindergartens, and the quiz, dance, and digital picture-story activities were acceptable for use with preschool children.

Teacher questionnaire responses were obtained from all 10 participating kindergartens (100% response rate) (Table 1 and Table S3). The survey results confirmed that the original DVDs could be screened without any issues at all kindergartens. In the questions directed at kindergarten teachers, the content that attracted the most interest from preschoolers was the “shokuiku (nutrition education) quiz” (Table 1).

### 3.2. Implementation Status of the Nutrition Educational Program

Based on the preliminary survey results, we incorporated “nutrition education quizzes,” “nutrition education dances,” and “digital picture-story shows” (Figure 1 and Figure 2), in which the children had shown a high level of interest. Notably, previous programs have utilized the dance [28]. During the intervention period, we successfully implemented all planned sessions across all nine kindergartens.

### 3.3. Comprehension Assessment

A total of 163 children (75 children in the inclusive group and 88 in the non-inclusive group) completed the assessment.

The overall distribution of scores (Table 2) showed 125 out of 163 children (76.7%) scored 3 or higher on dietary balance comprehension, and 122 children (74.8%) achieved a perfect score (2 points) on FA comprehension.

Table 3 summarizes the comprehension scores by educational setting and the results of the GEE analysis. After accounting for clustering within kindergartens, no significant association was found between the inclusive setting and the dietary balance score (Wald χ^2^(1) = 0.053, *p* = 0.818; OR = 0.912, 95% CI: 0.414–2.008). Although the median dietary balance score differed between groups (5.0 vs. 3.0), this outcome is a discrete scale ranging from 0 to 5 points and was heavily concentrated at the higher end; therefore, the median alone could not adequately characterize the overall ordinal distribution.

In contrast, an inclusive environment was significantly associated with higher FA comprehension scores (Wald χ^2^(1) = 4.750, *p* = 0.029; OR = 4.219, 95% CI: 1.156–15.399). The odds ratio (OR) for FA comprehension represents the cumulative odds that children in the inclusive group belong to a higher score category compared to those in the non-inclusive group.

### 3.4. Teacher Evaluations

Teachers reported relatively high VAS scores for the children’s concentration while watching the DVD (78.4 ± 16.0 points) and perceived effectiveness for nutrition education (68.6 ± 24.1 points), whereas the score for children discussing FA after watching the DVD was low (36.9 ± 31.4 points) (Table 4).

## 4. Discussion

The present study found that, following the implementation of the same standardized digital nutrition education program, preschoolers in an inclusive environment, where at least one peer with physician-diagnosed FA was present in the same class, had higher post-intervention FA comprehension scores than those in a non-inclusive environment. After accounting for clustering within kindergartens using GEE, the inclusive environment remained significantly associated with higher FA comprehension scores, whereas no significant association was observed for dietary balance.

### 4.1. Association Between Inclusive Environments and Understanding of FA

Following the digital nutrition education intervention, the inclusive environment was significantly associated with higher FA comprehension scores in the ordinal logistic GEE analysis (Wald χ^2^(1) = 4.750, *p* = 0.029; OR = 4.219, 95% CI: 1.156–15.399), whereas no significant association was observed for dietary balance score (Wald χ^2^(1) = 0.053, *p* = 0.818; OR = 0.912, 95% CI: 0.414–2.008). These results suggest that the association with the social environment may differ depending on the nature of the learning content. General nutritional knowledge, such as dietary balance, may be acquired through the visual and auditory information provided by digital educational materials, whereas FA-related knowledge may also be associated with recognition of peers’ dietary restrictions and consideration of others’ needs. From the perspective of observational learning and vicarious experience under social cognitive theory [15,29], preschoolers in an environment where children with FA are present may routinely observe allergy-conscious behaviors and the teachers’ responses during daily routines, such as lunchtime.

However, observational learning, peer interaction, and the frequency of FA-related experiences were not directly measured; therefore, this mechanism remains hypothetical. Future research is needed to clarify which aspects of the classroom or social environment may be associated with the observed differences in post-intervention FA comprehension.

### 4.2. Digital Instructional Materials

Our program received relatively high ratings in the teachers’ VAS evaluations for “children’s concentration” (78.4 points) and “effectiveness of nutrition education” (68.6 points) (Table 4). These ratings suggest that the instructional video appeared to effectively capture children’s attention and was perceived by teachers as a useful educational resource. Notably, teachers rated “whether the children discussed FA after watching the DVD” relatively low (36.9 points). One possible interpretation of the observed association with FA comprehension is that children in inclusive settings may have had opportunities for observational learning through daily experiences. Nevertheless, observational learning and relevant daily experiences were not directly assessed in the present study, and this interpretation therefore remains hypothetical. Previous studies have suggested that preschool children can engage in observational learning within social contexts [30,31], but further research is required to determine whether such processes contributed to the association observed in the present study.

### 4.3. Practical Implications and Application to Educational Methods

Our findings highlight both the challenges and potential of nutrition education in environments without peers with FA (non-inclusive settings). Digital teaching materials can provide standardized FA-related information; however, they may not capture the contextual experiences that occur in everyday classroom settings. Therefore, future research should examine whether additional contextual approaches—such as immersive storytelling, digital companion characters featuring FA, and role-playing—are associated with improved FA comprehension.

### 4.4. Limitations

This study has several limitations. First, regarding knowledge about food allergies, since no pre-intervention assessments were conducted, it is not possible to determine whether a difference in knowledge already existed between children in the inclusive and non-inclusive groups prior to the start of the digital nutrition education program or to draw causal inferences regarding any post-intervention differences. Second, due to practical constraints, the groups were defined based on existing classroom settings rather than through random assignment; consequently, the risk of selection bias and residual confounding remained. Third, although GEE was used in the present analysis to account for clustering of children within kindergartens, only nine kindergarten clusters were included, which may limit the precision of cluster-robust inference. Fourth, the binary definition of inclusive versus non-inclusive environment captured only the presence or absence of at least one peer with physician-diagnosed FA. It did not capture the number of children with FA, the frequency or nature of peer interactions, classroom organization, teacher guidance, or other FA-related practices. In addition, parental awareness of or communication about FA was not assessed. Therefore, differences in teacher practices or family-related factors may also have contributed to the observed association and cannot be distinguished from peer-related influences. Fifth, FA comprehension was assessed using only two study-specific items. These items were designed to correspond directly to the educational content and were reviewed for developmental appropriateness and comprehensibility, although they were not formally validated as a psychometric measure. The limited number of items may have restricted the precision of the assessment. Finally, the study was conducted among five-year-old children in nine public kindergartens within a single municipality in Hyogo Prefecture, which limits external validity and generalizability.

In order to elucidate the factors that deepen preschoolers’ understanding of FA, future research needs to incorporate not only methodological improvements, such as pre- and post-intervention assessments, validated outcome measures, and the monitoring of confounding factors, but also indicators that evaluate behavioral outcomes, such as the prevention of accidental ingestion and the sustained fostering of empathy toward peers with various health conditions.

## 5. Conclusions

This study found that the presence of a peer with FA in the same class was associated with higher post-intervention FA comprehension among preschool children who received a motivation-based digital nutrition education program. These findings suggest the potential value of considering social-environmental contexts in the design of digital nutrition education, with the broader goal of supporting nutritional literacy and prosocial awareness during early childhood. Further research is required to clarify the factors associated with preschoolers’ understanding of FA, including peer-, teacher-, and family-related factors.

## Figures and Tables

**Table 1:** Kindergarten teachers’ evaluation of children’s interest in nutrition educational activities during intervention development (n = 10).

Activity	Yes n (%)	Somewhat n (%)	No n (%)
Q1. Digital picture-story show	6 (60)	3 (30)	1 (10)
Q2. Quiz activity	10 (100)	0 (0)	0 (0)
Q3. Dance activity	9 (90)	1 (10)	0 (0)

Values are presented as n (%).

**Table 2:** Distribution of comprehension scores.

Food Balance Score				Food Allergies Comprehension Score			
Score	All	Inclusive Group ^a^	Non-Inclusive Group ^b^	Score	All	Inclusive Group ^a^	Non-Inclusive Group ^b^
	*n* (%)	*n* (%)	*n* (%)		*n* (%)	*n* (%)	*n* (%)
0	7 (4.3)	2 (2.7)	5 (5.7)	0	15 (9.2)	0 (0.0)	15 (17.0)
1	13 (8.0)	6 (8.0)	7 (8.0)	1	26 (16.0)	9 (12.0)	17 (19.3)
2	18 (11.0)	12 (16.0)	6 (6.8)	2	122 (74.8)	66 (88.0)	56 (63.6)
3	32 (19.6)	19 (25.3)	13 (14.8)	Total	163 (100.0)	75 (100.0)	88 (100.0)
4	0 (0.0)	0 (0.0)	0 (0.0)				
5	93 (57.1)	36 (48.0)	57 (64.8)				
Total	163 (100.0)	75 (100.0)	88 (100.0)				

^a^ presence of peers with food allergies. ^b^ absence of peers with food allergies.

**Table 3:** Comprehension scores by environmental group.

Outcome	Inclusive Group ^a^	Non-Inclusive Group ^b^	Wald χ^2^ (*df* = 1)	*p*	OR (95% CI)
Dietary balance score	3.6 ± 1.5; 3.0	3.9 ± 1.6; 5.0	0.053	0.818	0.912 (0.414–2.008)
FA comprehension score	1.9 ± 0.3; 2.0	1.5 ± 0.8; 2.0	4.75	0.029	4.219 (1.156–15.399)

Values are presented as mean ± standard deviation (SD) and median. OR = odds ratio; CI = confidence interval. ORs compare the inclusive group with the non-inclusive group (reference category). ^a^ Presence of at least one peer with a physician-diagnosed food allergy in the same class. ^b^ Absence of such a peer.

**Table 4:** Questionnaire results from kindergarten teachers (n = 32).

Item	Mean ± SD
Q1. Did the children watch the DVD attentively?	78.4 ± 16.0
Q2. Did the children talk about FA after watching the DVD?	36.9 ± 31.4
Q3. Do you think the DVD is effective in promoting nutrition education among children?	68.6 ± 24.1

Values are presented as mean ± standard deviation (SD).

## Data Availability

The raw data supporting the conclusions of this article will be made available by the authors on request due to ethical and privacy considerations.

## Supplementary Materials

The following supporting information can be downloaded at: https://www.mdpi.com/article/10.3390/nu18182972/s1, Table S1: Kindergarten teachers’ evaluation of children’s interest in nutrition education activities during the feasibility study. Table S2: Kindergarten teachers’ evaluations of children’s engagement and the perceived effectiveness of the food education program. Table S3: Questionnaire results of the pre-survey from kindergarten teachers (n = 10). Table S4: Questionnaire results of the pre-survey from kindergarten teachers Q5 (n = 10) Figure S1: Study design flow diagram. Figure S2: Japanese food guide spinning top.

## Abbreviations

The following abbreviations are used in this manuscript:

ARCS	Attention, Relevance, Confidence, and Satisfaction
Cl	Confidence interval
FA	food allergy
GEE	Generalized Estimating Equations
OR	Odds Ratio
VAS	Visual Analog Scale
