# Supplementary material for: Association Between Inclusive Educational Environments and Food Allergy Comprehension Among Preschool Children: A Quasi-Experimental Digital Intervention Study
Source: Nutrients. 2026 Sep 11;18(18):2972. doi: 10.3390/nu18182972 (PMC13610792; doi:10.3390/nu18182972)
Supplement: Supplementary file 1 [file nutrients-18-02972-s001.zip › nutrients-4518162-supplementary.pdf]

Supplementary Figure S1  Study design flow diagram

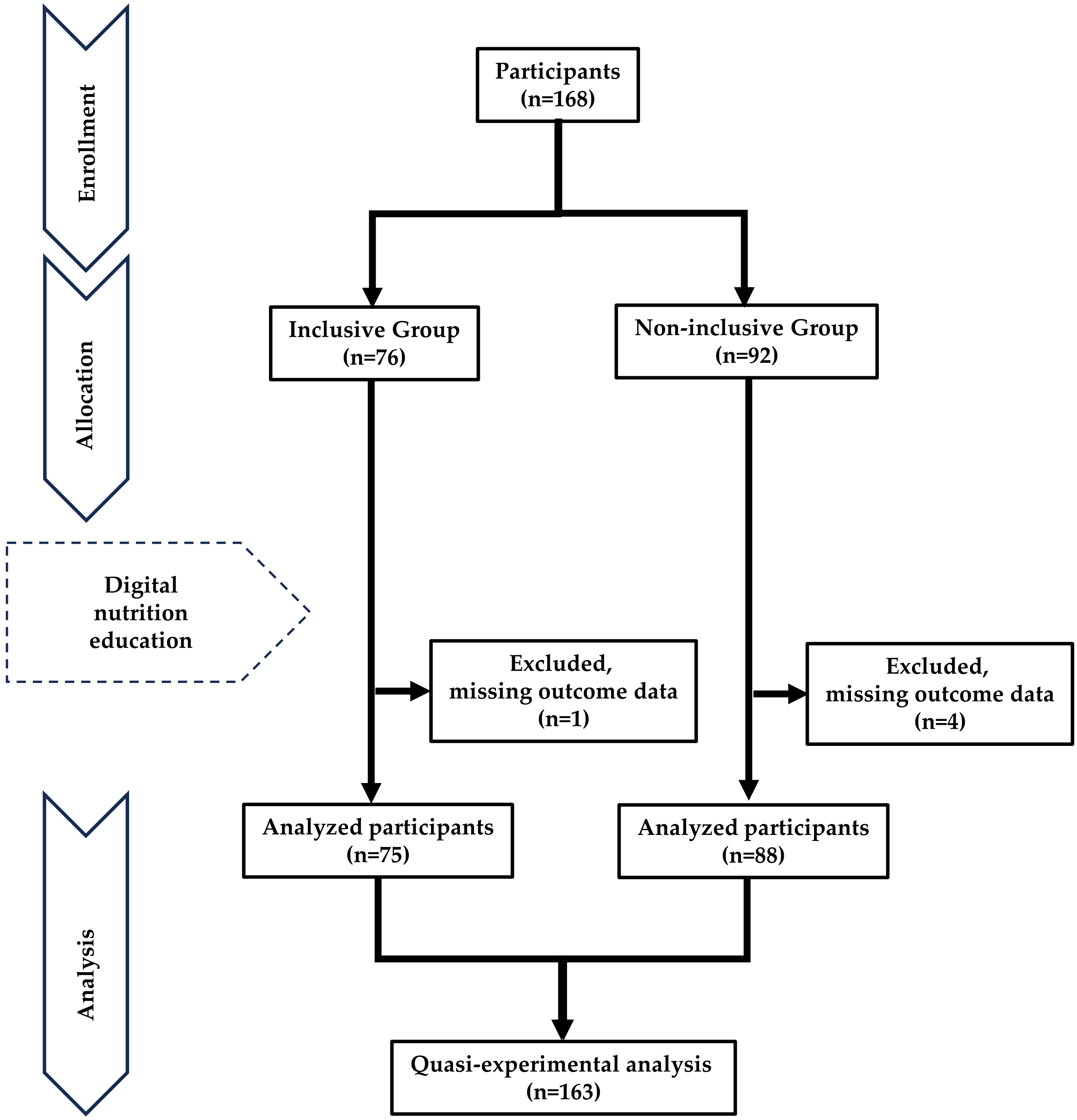

Supplementary Figure S2. Japanese Food Guide Spinning Top

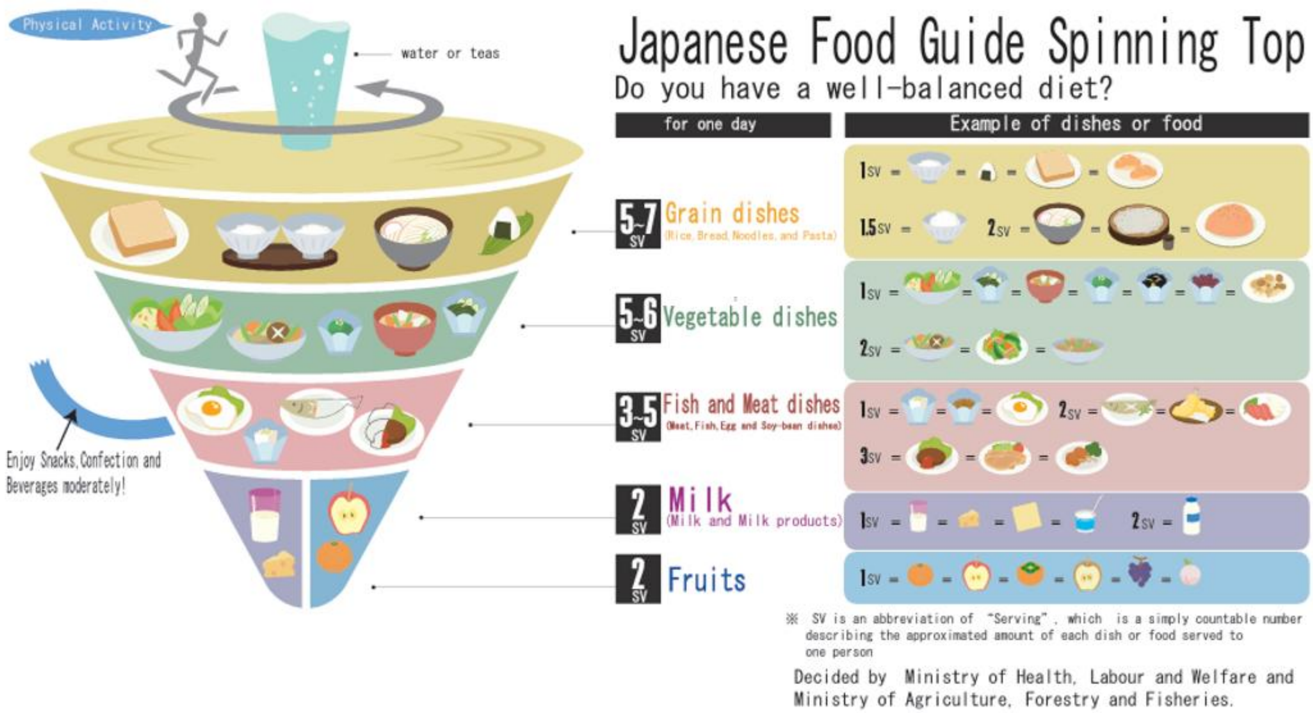

Table S1. Kindergarten teachers' evaluation of children's interest in nutrition education activities during the feasibility study.

Question 1

Did the digital picture-story show succeed in capturing the children's interest?

Question 2

Did the children answer the food education quiz?

Question 3

Did the children dance to the food education dance?

Question 4

What benefits do you expect from the "Food Education Delivery DVD"? (Multiple answers allowed)

1. Increased interest in food

2. Improvement of unbalanced diets

3. Spillover effects to the home

4. No expected benefits

5. Other

Question 5

What types of teaching materials or media would you like to use for children's food education? (Multiple selections allowed)

Table S2. Kindergarten teachers' evaluations of children's engagement and the perceived effectiveness of the food education program.

Question 1

Did the children watch the DVD attentively?

0 ———————————————————— 100

Not attentive at all                Very attentive

Question 2

Did the children talk about FA after watching the DVD?

0 ———————————————————— 100

Did not talk about it                Talked about it

Question 3

Do you think the DVD is effective in promoting nutrition education among children?

0 ———————————————————— 100

Not effective                Effective

Table S3. Questionnaire results of the pre-survey from kindergarten teachers (n = 10)

| Question | Increased interest in food | Improvement of unbalanced diets | Spillover effects to the home | No expected benefits | Other |
|---|---|---|---|---|---|
| Q4. Expected effects of the nutrition education DVD (Select all that apply) | 9 (90) | 3 (30) | 1 (10) | 0 (0) | 0 (0) |

Values are presented as n (%).

Table S4. Questionnaire results of the pre-survey from kindergarten teachers Q5 (n = 10)

| Question | CDs and DVDs | Visual storytelling materials | Hand games and dancing | Cooking and food handling | Online videos | External experts/specialists |
|---|---|---|---|---|---|---|
| Q5. Desired dietary education materials and media after the COVID-19 pandemic (Multiple responses) | 7 (70) | 7 (70) | 4 (40) | 4 (40) | 3 (30) | 2 (20) |

Values are presented as n (%).
